# Cognitive functioning in women with breast cancer: psychometric properties of the Arabic version of the Functional Assessment of Cancer Therapy-Cognitive Function Tool

**DOI:** 10.1186/s12955-023-02095-0

**Published:** 2023-01-28

**Authors:** Mariam Hassan, Zainab Barakat, Youssef Fares, Linda Abou-Abbas

**Affiliations:** 1 Neuroscience Research Center, Faculty of Medical Sciences, Lebanese University, Beirut, Lebanon; 2 INSPECT-LB (Institut National de Santé Publique Epidémiologie Clinique et Toxicologie-Liban), Beirut, Lebanon

**Keywords:** Breast cancer, Cognitive functioning, FACT-Cog, Validity, Reliability, Lebanon

## Abstract

**Background:**

The Functional Assessment of Cancer Therapy-Cognitive Function (FACT-Cog) evaluates perceived cognitive functioning and their impact on quality of life. This study was designed to evaluate the factors associated with cognitive functioning in a sample of women with breast cancer (BC) in Lebanon. We also sought to explore the psychometric properties of the FACT-Cog Arabic version.

**Methods:**

A cross-sectional study was carried out between March and August 2020 among women with BC. Socio-demographic and clinical characteristics were collected. In addition, patients were asked to complete the FACT-Cog Arabic version as well as the European Organization for Research and Treatment of Cancer Quality of Life Core Questionnaire 30, the Patient Health Questionnaire (PHQ-9), and the Generalized Anxiety Disorder (GAD-7). The internal consistency of the FACT-Cog tool was evaluated using Cronbach’s alpha. Content, convergent, and known group validity of the FACT-Cog Arabic version were also evaluated. All statistical analyses were performed using SPSS version 23.0.

**Results:**

A sample of 134 women with BC was collected. Internal consistencies of the FACT-cog total scale and its subscales were high (Cronbach’s α between 0.83 and 0.95). The convergent validity of the FACT-Cog Arabic version was supported by the positive correlation with the EORTC-cognitive functioning subscale. Moreover, negative correlations were found between FACT-Cog scale and fatigue, pain, anxiety, as well as depression. Known-group validity was supported by the statistically significant mean differences of the FACT-Cog total scale between patients in early (I &II) and late (III & IV) BC stages. Unmarried BC patients as well as those having higher depressive symptoms and a lower quality of life were found to be at higher risk of cognitive impairment.

**Conclusion:**

The FACT-Cog Lebanese Arabic version is a valid and reliable tool for assessing perceived cognitive functioning in BC women. Higher level of depression and impaired quality of life were associated with a decline in cognitive functioning.

## Introduction

Breast cancer (BC) is the most commonly diagnosed cancer in women and the second leading cause of cancer death in women globally [1, 2]. In BC patients, chemotherapy is the most commonly used treatment, which is often combined with radiation and surgery [3]. Due to advances in medical treatment, BC mortality rates have decreased in recent years, resulting in more survivors [4]. A prospective cohort study conducted among BC patients showed that chemotherapy decreased the patient's relative mortality risk by 25% and the risk of distant metastasis by 18% [5].

Despite improving cancer prognosis, chemotherapy has several physical and emotional side effects in BC women, including fatigue, nausea, vomiting, hair loss, hot flashes, anxiety, and distress [6]. Thus, BC is often no longer considered an incurable acute disease, but rather a chronic disease with remission periods and symptom exacerbation. This approach to BC has widened the scope of treatment from just treating the disease to controlling cancer-related symptoms like cognitive impairment (CRCI) [7]. Longitudinal studies indicated that approximately 30% of cancer patients experience CRCI before treatment begins, 75% have CRCI while undergoing active treatment (e.g., chemotherapy and hormonal therapy), and approximately 35% experience CRCI months or years after treatment is completed [8].

CRCI can be defined as the inability to remember, perform executive functions, concentrate, or pay attention [9]. CRCI is a major source of concern because it can impair treatment adherence, lower quality of life, and lead to long-term cognitive impairments [10]. In a study conducted to explore the types of cognitive changes noted in BC survivors, Von Ah et al. reported that BC survivors exhibited deficits in six major domains, including short-term memory, long-term memory, speed of processing, attention/concentration, language, and executive functioning [11]. It was also revealed that cognitive impairment has a significant impact on the self-perception, social network, and workability of breast cancer survivors [11]. Breast cancer patients are at high risk of developing CRCI, which negatively impacts their quality of life (QoL) [12]. Additionally, in longitudinal research conducted among breast cancer patients, authors concluded that patients with higher levels of anxiety and depression had greater cognitive impairment [13]. In another study, depression also predicted CRCI progression [14]. This assumes the presence of bidirectional correlations between cognitive complaints and some of their associated factors.

Cognitive assessment during the routine evaluation and care of cancer patients is essential. As a result, tool development and validation to systematically integrate cognitive screening into oncology clinical practice are highly recommended [15]. Such tools will assist clinicians and/or researchers in tracking and staging cognitive complaints and/or QoL in cancer patients. In response to this need, several objective and subjective tools have been developed, including neuropsychological tests, neuroimaging, and patient self-report. Despite its well-known benefits, direct neuropsychological testing is not always feasible or affordable [16]. As a result, patients are typically referred for neuropsychological testing only if they have noticed significant declines [16]. Furthermore, objective neuropsychological testing is time-consuming and requires a trained and qualified professional, which limits its use in practice [17]. Magnetic resonance imaging is also impractical for detecting cognitive impairment from cancer therapy in clinical settings [16]. Even though there are arguments against the validity of patients' self-reports, subtle declines in patients' cognitive function may be better detected through the use of this method than through neuropsychological testing [18]. Consequently, these limitations demonstrate the need for developing reliable and valid self-administered measures for assessing individuals' perceptions of their cognitive deficits, especially during the early stages of cognitive impairment [18].

The Functional Assessment of Cancer Therapy-Cognitive Function (FACT-Cog) version 3 is a questionnaire that assesses cognitive functioning and changes in quality of life [3]. This tool focuses on the noticeable interference on ability and function in the multiple specific domains related to perceived cognitive functioning [3]. The FACT-Cog questionnaire has been used to detect subjective cognitive deficits in cancer patients [19]. It was developed in English (USA) and has been cross-culturally adapted and validated in the following languages: French [20], Chinese [21], Korean [22], Turkish [23], Mexican Spanish [24], and Japanese [25]. However, no validated version of the Arabic language is currently available. As a result, translating, cross-culturally adapting, and validating the Arabic version of the FACT-Cog in patients with BC is essential to determine whether it can be used confidently as a reliable tool in future epidemiological studies and clinical trials.

This study aimed to evaluate the factors associated with cognitive functioning in a sample of Lebanese women with BC. We also sought to cross-culturally adapt and validate the FACT-Cog tool (version 3) in the Arabic language and evaluate its psychometric properties.

## Methods

### Cross-cultural adaptation of the FACT-Cog (version 3)

Permission has been requested from Mr. Jason BREDLE to translate the original FACT-Cog (version 3) questionnaire into the Arabic language. The standard Functional Assessment of Chronic Illness Therapy (FACIT) translation methodology was used to translate the FACT-Cog version 3. This iterative method was implemented and validated to verify that the translations are conceptually equivalent to the source content and that they are presented in a language that is culturally acceptable and relevant to the target audience. The translation and cross-cultural adaptation of the Fact-cog were performed according to the following five steps:

- *Step 1-Translation procedure of the FACT-Cog* two independent translators whose native language is Arabic had first translated the scale from English to Arabic. Translators were asked to avoid literal translation and to use a simple and acceptable language for the Lebanese population.
- *Step 2-Synthesis of the translations* the two translators synthesized the two translations. Discrepancies between the 2 independent forward translations have been discussed, and through a consensus process, an Arabic version of this tool has been produced.
- *Step 3-Back translation* one translator has carried out one back translation. The Back translation version and the initial English version were carefully compared by the first author (MH).
- *Step 4-Expert committee* an independent committee reviewed all the translated versions. An independent evaluation of the translated versions has been asked of each member of the panel, highlighting the linguistic, idiomatic, semantic, or cultural differences of each item in the questionnaire. Translation inconsistencies have been resolved by consensus, and a preliminary final version of the scale has been developed for field testing.
- *Step 5-Pre-test* the preliminary final version has been administered to a sample of 10 BC women who were asked to fill out the questionnaire. Then, interviews were conducted with the patients to discuss the meaning, comprehensibility, and acceptability of the items. The participants’ interview forms (PIF) were compiled and then evaluated by the study researchers.

The FACIT Measurement System research program coordinator checked the consistency between the final reconciled version and the back-translated English versions and provided comments to the study researchers, who then completed and chose the optimal translation. As needed, the translation was corrected.

### Study design and participants

A cross-sectional study was conducted between March and August 2020. Patients were recruited from Nabih Berry Governmental University Hospital (NBGUH), a public hospital in South Lebanon. A purposive sampling technique was used to select eligible participants. Women with histologically diagnosed BC, undergoing chemotherapy or hormonal therapy, aged 18 years or older, and who could read and understand Arabic (native language) were eligible to participate in our study. Patients were excluded from the study if BC was a second malignancy or if patients presented with medical conditions that might impair their cognitive functioning, such as brain metastasis, neuropsychiatric illness, or neurological disorder (dementia, multiple sclerosis, epilepsy, and Parkinson's disease). The medical records of the patients were reviewed to ensure that they have not received any neuropsychiatric or psychotropic drugs. All study participants provided written informed consent to participate.

### Sample size calculation

The sample size was computed based on results obtained from a previous study performed in South Korea [26]. The following parameters were used: an effect size (Cohen’s f2) of 0.10, an assumed two-sided significance of 5%, a power of 80%, and five predictors. This produced a total minimal sample size of 134 patients; the G-Power version 3.1.9.2 Kiel, Germany software was used for the sample size calculation.

### Ethical considerations

The study was reviewed and approved by the Neuroscience Research Center committee in the Faculty of Medical Sciences-Lebanese University. Researchers and fieldworkers conducted the study according to the research ethics guidelines laid down in the Declaration of Helsinki of the World Medical Association Assembly. Participation in the study was voluntary. Anonymity and information confidentiality were respected.

### Procedure

Eligible patients were informed about the study objectives. They were also informed that they have the right to withdraw from the study at any time. After receiving their written informed consent, participants were asked to complete a standardized questionnaire that included socio-demographic information, including age, marital status, and educational level. Clinical data, including previous medical history, cancer stage, type of treatment (chemotherapy and/or radiotherapy), and type of surgery (total or partial mastectomy), were collected from their medical files at the hospital. Then, a battery of tools was administered to patients, including the FACT-Cog scale, the European Organization for Research and Treatment of Cancer-Quality of Life Questionnaire-core (EORTC-QLQ-C30), the Patient Health Questionnaire (PHQ-9), and the Generalized Anxiety Disorder (GAD-7).

### Study measurements

*The FACT-Cog (version 3)* is a self-reported 37-item questionnaire that consists of four subscales: Perceived Cognitive Impairment-CogPCI (20 items), Perceived Cognitive Ability-CogPCA (9 items), Comments from Others on Cognitive Function-CogOth (4 items), and Impact on Quality of Life-CogQoL (4 items). All items are rated for the previous week, including the day of administration, on a 5-point Likert scale ranging from 0 “Never” or “Not at all” to 4 “Several times a day” or “Very much”. The total CogPCI subscale ranges from 0 to 72, the CogPCA ranges between 0 and 28, and each of the Cog-QoL and Cog-Oth ranges between 0 and 16. The total score for the FACT-Cog is computed by summing all the item scores and ranges from 0 to 148 points, with a higher score indicative of better perceived cognitive functioning. The sum of the individual item scores of each subscale (except for Cog-MT1 and MT2 that belong to the CogPCI and Cog-PMT1 and PMT2 that belong to the CogPCA) yields the composite score, and higher scores indicate better cognitive functioning.

*The European Organization for Research and Treatment of Cancer Quality of life Core Questionnaire 30 (EORTC-QLQ-C30)* is a questionnaire developed and validated to assess the QoL. This tool is composed of five multi-item functional scales (physical, role, cognitive, emotional, and social), three multi-item symptom scales (fatigue, pain, and nausea/vomiting), five single items (dyspnea, insomnia, appetite loss, constipation, diarrhea), and a global health status (2 items) [27]. Items of the functional and symptom scales were assessed using a 4-point Likert scale ranging from “Not at all” to “Very much”. The two items of the global health status/quality of life scale have response options ranging from [1] “very poor” to [7] “excellent”. As indicated in the scoring manual, all scores were transformed linearly into a range from 0 to 100. A higher score on the functional scale indicated a better level of functioning, a higher score on the global health status scale indicated higher QoL, and a higher score on the symptom scale indicated a worse level of symptomatology. The Arabic version of the EORTC-QLQ-C30 was previously validated in patients with cancer among the Lebanese population [28].

*The Patient Health Questionnaire (PHQ-9)* is a screening tool used to assess the severity of depressive symptoms. Each item of the PHQ-9 is scored on a 4-point Likert scale, ranging from 0 (not at all) to 3 (nearly every day). The PHQ-9 total score ranges from 0 to 27, with higher scores indicating a higher level of depression [29]. The PHQ-9 was translated into Arabic and validated among the Lebanese population [30].

*The Generalized Anxiety Disorder-7 (GAD-7)* is a 7-item screening tool used to assess the severity of anxiety symptoms. Each item of the GAD-7 is scored on a 4- point Likert scale ranging from 0 (not at all) to 3 (nearly every day). The GAD-7 total score ranges from 0 to 21, with a higher score indicating a higher level of anxiety. The GAD-7 was translated into Arabic and validated among the Lebanese population [30].

### Statistical analysis

All analyses were performed using SPSS (version 23.0 for Windows). Descriptive statistics were reported using means and standard deviations (SD) for continuous variables and frequency with percentages for categorical variables. Cronbach's alpha was calculated to assess the reliability of the FACT-Cog. Convergent validity was assessed by correlating FACT-Cog with EORTC-Cognitive Functioning subscale (EORTC-CF), PHQ-9, GAD-7, fatigue, and pain. A correlation coefficient value of 0.70 and above indicated a strong correlation, 0.40 to 0.70 indicated a moderate correlation, and values ranging from 0 to 0.40 indicated a weak correlation [31]. Known-group validity was evaluated by examining the scale ability to discriminate between two groups of patients differing in disease stage (stages I & II vs. stages III & IV). Independent t-tests were calculated to investigate potential differences in the mean scores of the FACT-Cog scale between the two groups. The effect size of the statistically significant difference was calculated using Cohen’s D. A Cohen D of 0.2 is indicative of small effect size, 0.5 a medium effect size, and 0.8 or higher as large effect size [32]. Finally, a multiple linear regression analysis was conducted to explore factors associated with the FACT-Cog total scale. Assumptions of the model adequacy for linear regression linearity; normality of distribution and multicollinearity were assessed. Unstandardized beta (β) coefficients with 95% confidence intervals (CI) and standardized beta were reported. In all statistical analyses, a P value of less than 0.05 is considered significant.

## Results

### Baseline characteristics of the study participants

A total of 146 women with BC were invited to participate of whom 135 gave their verbal consent to participate (response rate 92.5%). Several reasons were reported by the 11 women who refused to participate such as poor health or pain, tense psychological conditions, cessation of treatment, shame or embarrassment, and non-acceptance from family members.

Table 1 summarizes the demographic and clinical characteristics of the breast cancer women who participated in the study. The mean age of the respondents was 52 years old (SD = 9.95), ranging from 32 to 73 years. The majority (71.6%) were married. Regarding BC stages, approximately one-third of the participants (27.6%) were diagnosed with Stage I, a third (34.3%) with Stage II, and another third (38.1%) with Stages III and IV. Concerning treatment, 103 (76.9%) patients underwent surgery, of which 85 (63.4%) had a total resection. Plus, the majority of the patients (94.8%) were treated with chemotherapy, and approximately half of the sample (50.8%) underwent radiotherapy.

**Table 1:** Sociodemographic and clinical characteristics of the study sample

Variables	Patients (N = 134)
Age (mean ± SD)	52.05 ± 9.95
*Marital status n (%)*	
Single	19 (14.2%)
Married	96 (71.6%)
Divorced	11 (8.2%)
Widowed	8 (6%)
*Level of Education n (%)*	
Primary	34 (25.4%)
Complementary	50 (37.3%)
Secondary	28 (20.9%)
University and above	22 (16.4%)
*Comorbidities n (%)*	
No	78(58.2)
Yes	56(41.8)
*Stage of Cancer n (%)*	
Stage I	37 (27.6%)
Stage II	46 (34.3%)
Stage III & IV	51 (38.1%)
*Type of Mastectomy n (%)*	
Total	85 (63.4%)
Partial	18 (13.4%)
*Treatment n (%)*	
Chemotherapy	127 (94.8%)
Radiation	68 (50.8%)

*N* frequency; *%* percentage; *SD* standard deviation

### Descriptive statistics of the FACT-Cog total scale and its subscales

The mean of the FACT-Cog total scale was 83.0 (SD = 21.5). No significant floor and ceiling effects were detected as the proportion of patients reaching the minimum and maximum scores were 0.7%. Means and SDs of the FACT-cog subscales are reported in Table 2.

**Table 2:** Internal consistency of the Arabic version of the FACT-Cog (N = 134)

	Mean (SD)	Scale mean if item deleted	Scale variance if item deleted	Corrected item-total correlation	Cronbach's Alpha if Item deleted	Cronbach's Alpha
FACT-Cog Total scale	83(21.5)					0.92
Perceived Cognitive Impairments	47.5(12.5)					0.92
CogA1		18.38	172.10	0.76	0.917	
CogA3		18.33	171.74	0.75	0.917	
CogC7		17.56	166.72	0.74	0.917	
CogM9		18.90	185.05	0.43	0.924	
CogM10		17.17	172.35	0.64	0.919	
CogM12		17.42	167.52	0.70	0.918	
CogV13		18.64	179.20	0.58	0.921	
CogV15		18.75	179.84	0.60	0.921	
CogV16		18.72	181.07	0.55	0.922	
CogV17b		18.74	180.64	0.56	0.922	
CogF19		17.24	171.64	0.62	0.920	
CogF23		17.93	168.71	0.70	0.918	
CogF24		17.01	171.69	0.46	0.926	
CogF25		18.17	171.66	0.59	0.921	
CogC31		17.84	168.97	0.69	0.918	
CogC32		18.37	172.05	0.79	0.916	
CogC33a		18.39	176.48	0.46	0.924	
CogC33c		18.42	176.45	0.56	0.921	
CogMT1		20.09	216.44	0.64	0.928	
CogMT2		20.11	216.07	0.65	0.928	
Perceived Cognitive Abilities	13.2(3.54)					0.92
CogPC1		22.04	51.42	0.67	0.918	
CogPV1		21.41	54.46	0.57	0.923	
CogPM1		22.38	51.65	0.71	0.915	
CogPM2		22.05	50.64	0.73	0.913	
CogPF1		21.78	51.20	0.75	0.912	
CogPCH1		21.74	50.93	0.76	0.912	
CogPCH2		22.27	50.32	0.74	0.913	
CogPMT1		21.61	49.87	0.78	0.910	
CogPMT2		21.62	49.66	0.78	0.910	
Comments from Others	14.5(4.80)					0.83
CogO1		1.58	6.87	0.55	0.861	
CogO2		2.41	8.03	0.68	0.784	
CogO3		2.29	7.48	0.78	0.738	
CogO4		2.21	7.41	0.70	0.768	
Impact On Quality Of Life	7.80(5.24)					0.95
CogQ35		6.16	16.30	0.79	0.971	
CogQ37		6.02	15.76	0.92	0.930	
CogQ38		6.20	15.56	0.92	0.931	
CogQ41		6.18	15.31	0.93	0.929	

*N* frequency, *SD* standard deviation

### Reliability of the FACT-Cog subscales

The Cronbach’s alpha coefficient for the Arabic version of the FACT-Cog was 0.93. All subscales showed a high level of consistency: CogPCI (alpha = 0.92), CogPCA (alpha = 0.92), CogOth (alpha = 0.83), and CogQoL (alpha = 0.95). All items-total correlations were greater than 0.30, indicating that all items contributed adequately to the corresponding subscale (Table 2).

### Validation of the FACT-Cog scale Arabic version

#### Face and Content Validity

To check the clarity and appropriateness of the target language version, the final Arabic FACT-Cog version was piloted on 10 patients. The questionnaire was completed in 10–12 min. The PIFs were evaluated to determine whether patients felt difficulties or ambiguity in responding to the items. BC patients indicated that the questionnaire was acceptable.

#### Convergent Validity

A moderate correlation was found between the total score of the Arabic version of the FACT-Cog and the score of the EORTC-CF (r = 0.640, P value < 0.0001). There were also moderately significant correlations between all subscales of the Arabic version of the FACT-Cog and the EORTC-CF (r = 0.488 to 0.566, P value < 0.0001). A moderate negative correlation was found between the FACT-Cog total score and the PHQ-9 (r = − 0.660, P value < 0.0001), suggesting evidence for convergent validity. There were also significantly moderate negative correlations between the four FACT-Cog subscales and the PHQ-9 scale (r = − 0.502 to − 0.564, P value < 0.0001). The convergent validity of the FACT-Cog total score and the four FACT-Cog subscale scores with GAD-7, fatigue, and pain were also evaluated; weak to moderately significant negative correlations were found (Table 3).

**Table 3:** Convergent validity of the FACT-Cog total scale and its domains

	FACT-Cog total	CogPCA	CogPCI	CogOth	CogQoL
EORTC-CF	0.640**	0.526**	0.488**	0.526**	0.566**
PHQ-9	− 0.660**	− 0.540**	− 0.564**	− 0.502**	− 0.545**
GAD-7	− 0.388**	− 0.296**	− 0.248*	− 0.356**	− 0.369**
Fatigue†	− 0.409**	− 0.296**	− 0.248*	− 0.356**	− 0.321*
Pain††	− 0.331**	− 0.236*	− 0.271*	− 0.321*	− 0.408**

FACT-Cog Functional Assessment of Cancer Therapy-Cognitive Function, CogPCA: perceived cognitive abilities subscale; CogPCI: perceived cognitive impairment subscale; CogOth: comments from others subscale; CogQoL: quality of life subscale; EORTC-CF Cognitive Functioning scale, PHQ-9 Patient Health Questionnaire, GAD-7 Generalized Anxiety Disorder,

^†^ Fatigue evaluated by the EORTC-QLQ C30 scale

^††^ Pain evaluated by the EORTC-QLQ C30 scale

*P value < 0.05

**P value < 0.0001

#### Known-group validity

Comparison of the FACT-Cog total and its subscales mean values between patients in early (I &II) and late (III & IV) BC stages are presented in Table 4. BC women in late BC stages rated worse functioning compared to those in early stages in the FACT-cog total scale and all its subscales (P < 0.05) except the quality of life subscale. ES ranged between 0.09 and 0.59, with the perceived cognitive impairment subscale displaying the highest ES.

**Table 4:** Mean scores of the FACT-Cog total scale and its domains in two groups of patients differing by disease stage (stage I&II compared to stage III-IV)

	Group 1 (Stage I & II) Mean (SD)	Group 2 (Stage III & IV) Mean (SD)	P value	Effect size
FACT-Cog Total	87.6 (17.1)	75.5 (25.8)	0.004	0.55
CogPCA	15.3(4.4)	13.2(5.1)	0.015	0.44
CogPCI	50.4(10.3)	42.9(14.5)	0.002	0.59
CogOth	13.9(2.7)	11.9(4.3)	0.003	0.56
CogQoL	8.0(5.1)	7.5(5.5)	0.564	0.09

FACT-Cog Functional Assessment of Cancer Therapy-Cognitive Function, CogPCA: perceived cognitive abilities subscale; CogPCI: perceived cognitive impairment subscale; CogOth: comments from others subscale; CogQoL: quality of life subscale; SD Standard deviation, higher mean scores indicates better perceived cognitive functioning, P < 0.05 is considered significant

### Factors associated with the FACT-Cog total scale

A multivariable linear regression analysis was conducted to explore the predictors of the FACT-Cog total score (Table 5). PHQ-9, EORTC-CF, and marital status were the statistically significant predictors of the FACT-Cog total score (standardized beta = − 0.433, 0.304, and − 2.014, respectively; all P values < 0.05). The total variance explained by the linear regression model was 51.6%.

**Table 5:** Factors associated with the FACT-Cog total score in the patient group

	Unstandardized coefficients (B)	Standardized coefficients (B)	95% confidence Interval	P value
PHQ-9	− 1.976	− 0.433	− 2.827–(− 1.126)	˂0.0001
EORTC-CF	0.683	0.304	0.267–1.098	0.001
Marital Status	− 5.953	− 2.014	− 11.764–(− 0.105)	0.046

FACT-Cog Functional Assessment of Cancer Therapy-Cognitive Function, PHQ-9 Patient Health Questionnaire, GAD-7 Generalized Anxiety Disorder-7, EORTC-CF Cognitive Function, Variables entered to the model: age, marital status, education, comorbidities, Chemotherapy (Yes/No), Radiotherapy (Yes/No), Mastectomy (yes/No), PHQ-9, GAD-7, EORTC-CF

Adjusted R^2^ = 0.516, p < 0.05 is considered significant

## Discussion

The current study was conducted to evaluate the factors associated with cognitive functioning and to examine the psychometric properties of the FACT-Cog scale Arabic version in a sample of Lebanese women with BC. Our results showed that the Arabic version of the FACT-Cog tool can be used to assess cognitive functioning in Arabic-speaking individuals and has good psychometric properties. Higher depression level, impaired quality of life, as well as being unmarried, were found to be predictors of cognitive impairment.

In our study, all FACT-Cog subscales demonstrated acceptable internal consistency, including CogPCI (α = 0.92), CogPCA (α = 0.92), CogOth (α = 0.83), and CogQoL (α = 0.95). These results support the appropriate reliability of this version and are similar to those obtained from the French [20], Chinese and English [33], Korean [3], and Japanese versions [34]. Besides, the Cronbach’s alpha coefficient for the CogOth subscale is clearly lower than that of the other subscales, which is probably due to the smaller number of items in this subscale. This finding is in agreement with the results reported by the Japanese (α = 0.73) [34], French (α = 0.70) [20], and Korean versions of the FACT-Cog scale (α = 0.84) [3].

Concerning face and content validity, our participants did not report any problems in understanding the scale. Therefore, no further changes to the scale were made. Regarding convergent validity, a moderate correlation was observed between the FACT-Cog Arabic version total score and the EORTC-CF subscale (r = 640, P value < 0.0001). This finding is in line with the relationship found between the EORTC-CF and the FACT-Cog total score in the Chinese version [33]. Furthermore, our results demonstrated adequate validity for the four FACT-Cog subscales. Each subscale of the Arabic version of the FACT-Cog (PCI, PCA, Oth, and QoL) has a moderately significant relationship with the EORTC-CF (r = 0.488, 0.526, 0.526, and 0.566, respectively). This finding is in accordance with the significantly moderate correlation obtained between the Korean version of FACT-Cog and the EORTC-CF [3]. Moreover, a moderate negative correlation between the FACT-Cog total score and depression (r = − 0.660, P value ˂ 0.0001) was found. There were also significantly weak to moderate inverse correlations between FACT-Cog subscale scores and patients’ pain, fatigue, anxiety, and depression. As expected, higher levels of cognitive complaints were associated with higher levels of depression, anxiety, fatigue, and pain. These findings support the convergent validity of the Arabic version of the FACT-Cog scale; they are in line with previous research that showed significant correlations between cognitive complaints and psychosocial factors [3, 33]. It is important to note that, due to the multifactorial nature of cognitive impairment, strong correlations with the discussed confounders would be difficult to obtain. As a result, no single attribute is sufficient to generate a strong correlation with higher perceived cognitive impairments.

Known-group validity was demonstrated with statistically significant better FACT-Cog reported by patients in early stage compared to those with late stages of the disease. Thus, the FACT-cog total scale was able to successfully discriminate between early and late stages of cancer among women, such that patients with advanced disease revealed higher cognitive impairments than those with early stages of the disease. Furthermore, ES showed that the detected statistically significant differences were clinically relevant.

Multivariable linear regression analysis revealed that depression, QoL, and marital status were associated with cognitive function in BC patients. Higher depressive symptoms were found to be associated with impaired cognitive functioning. This finding is in line with the one obtained in a study conducted among middle-aged and elderly populations to determine the longitudinal association between depressive symptoms and cognitive performance [35]. It is worth mentioning that several studies have found that depression is very common among breast cancer patients and is associated with significant functional impairment and decreased survival rate [36–38]. This emphasizes the importance of regular psychological assessment in BC patients, as well as the need for early intervention to postpone the onset of cognitive deficits. Besides, cognitive complaints were found to be associated with a lower QoL. Consistent with our findings, Von Ah et al. [39] reported significant subjective cognitive impairment in younger BC survivors, which is related to greater decrements in some QoL outcomes, such as physical functioning and fatigue. In addition, our study showed that unmarried women had higher levels of cognitive impairment as compared to their married counterparts. This could be attributed to the poor health, as well as the various sensitive stages of diagnosis and treatment that the BC patients are undergoing, where they solely require a truly supportive partner who could improve their health, including cognition. This result is inconsistent with the study conducted to investigate the relationship between marital status and cognitive impairment among community-dwelling Chinese older adults, which found a significant association between marital status and cognitive impairment in men, but not in women [40].

The present research has some limitations. First, the test–retest reliability and the construct validity of the scale were not assessed. Future longitudinal studies with a larger sample are necessary to evaluate changes in perceived cognitive functioning over time and confirm the construct validity of the scale in the Lebanese population. Second, while QLQ-30 is not the perfect standard for assessing the concurrent validity of the FACT-Cog, it was used because it is the only validated questionnaire available for evaluating all the domains of cognitive functioning in cancer patients. Third, because of the cross-sectional nature of the study, it is not possible to ascertain the reverse causality or temporal relationship concerning the pathways of association between cognitive functioning and various associated factors which are likely to be bidirectional. Finally, since this scale has only been validated in Arabic among breast cancer women, it needs to be validated among breast cancer men and other patients with different types of cancer before it can be used in oncology clinical settings.

## Conclusion

The Arabic version of the Fact-Cog scale is a valid and reliable self-report measure for assessing perceived cognitive impairment in Lebanese breast cancer women. Using the Fact-Cog scale, healthcare workers in Arab countries can assess the cognitive function of BC patients during and after treatment. According to our study, cognitive decline is associated with higher levels of depression and impaired quality of life. This suggests developing supportive care intervention programs in this population to reduce the likelihood of negative cognitive outcomes.

## Data Availability

The dataset analyzed during the current study are available from the corresponding author on reasonable request.

## Abbreviations

FACT-Cog: Functional Assessment of Cancer Therapy-Cognitive Function
BC: Breast cancer
CogPCA: Perceived cognitive abilities subscale
CogPCI: Perceived cognitive impairment subscale
CogOth: Comments from others subscale
CogQoL: Quality of life subscale
EORTC-QLQ-C30: European Organization for Research and Treatment of Cancer Quality of Life Core Questionnaire 30
EORTC-CF: Cognitive functioning scale
SPSS: Statistical package for social sciences
FACIT: The standard functional assessment of chronic illness therapy
PHQ-9: Patient Health Questionnaire
GAD-7: Generalized anxiety disorder
CI: Confidence interval
SD: Standard deviation
QoL: Quality of life
